# New Method of Analysis of Lipids in *Tribolium castaneum* (Herbst) and *Rhyzopertha dominica* (Fabricius) Insects by Direct Immersion Solid-Phase Microextraction (DI-SPME) Coupled with GC–MS

**DOI:** 10.3390/insects10100363

**Published:** 2019-10-19

**Authors:** Ihab Alnajim, Xin Du, Byungho Lee, Manjree Agarwal, Tao Liu, Yonglin Ren

**Affiliations:** 1 College of Science, Health, Engineering and Education, Murdoch University, Perth 6150, Australia; alnajim2015@yahoo.com.au (I.A.); B.Du@murdoch.edu.au (X.D.); Y.Ren@murdoch.edu.au (Y.R.); 2 Date Palm Research Centre, University of Basrah, Basra 61004, Iraq; 3 Research Institute of Life Science, GyeongSang National University, Jinju 52828, Korea; byungholee@hotmail.com; 4 Institute of Equipment Technology, Chinese Academy of Inspection and Quarantine, No. A3, Gaobeidianbeilu, Chaoyang District, Beijing 100123, China

**Keywords:** DI-SPME, GC–MS, insect lipids, insect hydrocarbons, *T. castaneum*, *R. dominica*

## Abstract

Lipids play an essential role in providing energy and other physiological functions for insects. Therefore, it is important to determine the composition of insect lipids from cuticular and internal tissues for a better understanding of insect biology and physiology. A novel non-derivatization method for the analysis of lipids including fatty acids, hydrocarbon waxes, sterols in *Tribolium castaneum* (Herbst) and *Rhyzopertha dominica* (Fabricius) was explored using the direct immersion solid-phase microextraction (DI-SPME) coupled with gas chromatography–mass spectrometry (GC–MS). Nine extraction solvents, acetonitrile, methanol, hexane, ethanol, chloroform, acetonitrile and ethanol (1:1 *v*/*v*), acetonitrile and water (1:1 *v*/*v*), ethanol and water (1:1 *v*/*v*) and acetonitrile and ethanol and water (2:2:1 *v*/*v*/*v*) were selected and evaluated for the extraction of insect lipids with DI-SPME fiber. Acetonitrile extraction offered the best qualitative, quantitative, and number of lipids extracted from insects samples results. Acetonitrile extracted high-boiling point compounds from both species of tested insects. The range of hydrocarbons was C25 (pentacosane) to C32 (dotriacontane) for *T. castaneum* and C26 (11-methylpentacosane) to C34 (tetratriacontane) for *R. dominica*. The major compounds extracted from the cuticular surface of *T. castaneum* were 11-methylheptacosane (20.71%) and 3-methylheptacosane (12.37%), and from *R. dominica* were 10-methyldotriacontane (14.0%), and 15-methyltritriacontane (9.93%). The limit of detection (LOD) for the n-alkane compounds ranged between 0.08 (nonacosane) and 0.26 (dotriacontane) µg/g and for the fatty acids between 0.65 (arachidic acid) to 0.89 (oleic acid) µg/g. The study indicated that DI-SPME GC–MS is a highly efficient extraction and a sensitive analytical method for the determination of non-derivatized insect lipids in cuticular and homogenized body tissues.

## 1. Introduction

Lipids are the main biological compounds in animals and plants [1], including fatty acids and hydrocarbon waxes on and in insect bodies [2]. Fatty acids are the most basic form of biological lipids and are usually bound with other compounds to build more composite lipids, such as triglycerides, which are energy stores [3]. The cuticular lipid layer of the insect consists of different chemicals such as a long chain of hydrocarbons and fatty acids [4]. An analysis of the cuticular lipids of *Acanthoscelides obtectus* showed that adults contain a variety of chemicals such as hydrocarbons, triacylglycerols, fatty acid esters, free fatty acids, sterols, aldehydes, ketones and alcohols [5]. Hydrocarbons are the major lipid category in the cuticle of insects, including straight-chain saturated and unsaturated hydrocarbons [6], which in some species reach to more than 90% of cuticular lipids and are usually a mixture of components including n-alkanes, branched methyl-alkanes and ethyl-alkanes [2]. An analysis of cuticular lipids from adults of *Zygogramma exclamationis* showed that large amounts of lipids in the cuticle of males and females were hydrocarbons ranging from C23 (tricosene) to C56 (trimethyltripentacontane) [7]. The functions of insect cuticular hydrocarbons have evolved to not only keep water in the organism but to also play an essential role in communication among the population individuals and between individuals of different sexes [2,8,9]. A study on the adults of *Drosophila melanogaster* demonstrated that increased desiccation resistance is linked with the total amount of cuticular hydrocarbons [10]. Cuticular lipids have also been reported to protect insects from harmful pathogens [2,11]. The composition of cuticular lipids varies among the insect species, and it is a reflection of the genetic structure and changes induced by ecological circumstances [12]. Therefore, an analysis of insect lipids is fundamental to understand insect metabolism and physiology.

The extraction of lipids from insects is the first critical step that leads to a reliable qualitative and quantitative analysis of insect lipids [1]. Numerous methods have been used to extract insect lipids; however, extraction solvents and methods are the core of a reliable extraction process that transfers lipids from a matrix to the liquid phase [1,13,14,15], thus enabling coupling with various analytical methods [1]. Examples include using a single solvent like hexane to extract cuticular hydrocarbons from six species of flies and an analysis with gas chromatography–mass spectrometry (GC–MS) [13], extraction with chloroform and an analysis with high-performance thin-layer chromatography (HPTLC) to determine surface lipids of *Bemisia argentifolii* [14], and extraction with dichloromethane and an analysis with gas chromatography–Flame ionization detector (GC–FID) for profiling the cuticular hydrocarbons of four *Periplaneta* species including *Periplaneta brunnea*, *Periplaneta fuliginosa*, *Periplaneta australasiae* and *Periplaneta americana* [15]. Commonly, the use of two solvents, such as hexane/chloroform, have been used for extraction of the cuticular lipids from the *Osmia lignaria* and *Megachile rotundata* bees and the *Aleurodicus dugesii* giant whitefly, which were then analyzed with GC–FID and GC–MS [16].

Solid-phase microextraction (SPME) has been used to extract insect lipids, especially cuticular lipids [1]. This extraction is done either by rubbing the SPME fiber onto the surface of the insect cuticle [17,18] or by using the headspace solid-phase microextraction (HS-SPME) method [19]. However, these procedures are not suitable for the analysis of semi-volatile compounds. Keeping this in mind, a novel method of direct immersion solid-phase microextraction (DI-SPME) in different solvents coupled with GC–MS has been used. Therefore, this study aimed to evaluate the use of the DI-SPME technique coupled with GC–MS to extract and analyze insect lipids like fatty acids and hydrocarbon waxes from the surface and whole adult body of two main stored products insects, *Tribolium castaneum* and *Rhyzopertha dominica.* This technique involves direct immersion in the solvents, so the appropriate solvent selection is critical. Therefore, a range of solvents including acetonitrile, methanol, hexane, ethanol, chloroform, acetonitrile and ethanol (1:1 *v*/*v*), acetonitrile and water (1:1 *v*/*v*), ethanol and water (1:1 *v*/*v*), and acetonitrile and ethanol and water (2:2:1 *v*/*v*/*v*) were evaluated in this study in order to determine their ability to enhance the extraction of the analytes.

## 2. Material and Methods

### 2.1. The Insect Culture

Adult insects, *Tribolium castaneum* and *Rhyzopertha dominica,* were obtained from the Department of Primary Industries and Regional Development (DPIRD), Australia. The work with the two species of insects was approved by Murdoch University (Approval number: WBM-18-249), as there was no ethical concern about using *T. castaneum* and *R. dominica*. The narrow aged insects (2–3 days) were cultured by incubating 3000 adult insects with 1000 g of food—broken wheat for *R. dominica* (strain MUWRD-7), and wheat flour/yeast (12:1) ratio for *T. castaneum* (strain MUWTC-6000) in 2-L jars sealed with meshed lids. The parents’ insects were removed after three days, and the remaining culture was incubated at 28 ± 1 °C and 70 ± 2% relative humidity (RH). Newly emerged adults were narrowly aged and transferred to the jar containing new food. The insects used in the experiments were one month old. The flour was made from freshly harvested wheat (Australian Standard Wheat). Before use, the wheat was sterilized by keeping it at −20 °C for seven days, followed by storage at 4 °C until use. The grain was milled using a Wonder Mill (Model WM2000, WonderMill, Korea), and the flour was kept at 4 °C.

### 2.2. Chemical Reagents and Equipment Used

The solvents used were acetonitrile ≥99.9% *v*/*v* (Fisher Scientific, Glee, Belgium), methanol ≥99.9% *v*/*v*, hexane ≥95% *v*/*v*, ethanol ≥99.9% *v*/*v*, and chloroform ≥99.9% *v*/*v* (Sigma-Aldrich, Bellefonte, PA, USA). Deionized water (DI) was purified through a Milli-Q Biocel system (Millipore, Burlington, MA, USA).

Various combinations of mixed solvents were prepared and used, such as acetonitrile and ethanol (1:1 *v*/*v*), acetonitrile and water (1:1 *v*/*v*), ethanol and water (1:1 *v*/*v*), and acetonitrile and ethanol and water (2:2:1 *v*/*v*/*v*).

Individual external standards were purchased from Merck-Sigma Aldrich Co. and included palmitic acid ≥99% *w*/*w*, linolenic acid ≥99% *w*/*w*, linoleic acid ≥99% *w*/*w*, oleic acid ≥99% *w*/*w*, arachidic acid ≥99% *w*/*w*, cholesterol ≥99% *w*/*w*, p-benzoquinone ≥98% *w*/*w*, 2-methyl-p-benzoquinone ≥98% *w*/*w* and 1-pentadecene ≥98% *v*/*v*. In addition, n-alkane standard C7–C40 (1000 mg/mL in hexane) was purchased from (Supelco, Bellefonte, PA, USA).

The 50/30 µm divinylbenzene/carboxen/polydimethylsiloxane (DVB/CAR/PDMS) SPME fiber with a 2 cm coating was supplied by Sigma-Aldrich, Bellefonte, PA, USA. Prior to use, the fiber was activated according to the manufacturer’s recommendations by exposing the fiber’s coating to 270 °C for half an hour.

### 2.3. GC–MS Instrument and Analytical Conditions

All GC–MS analyses were performed with an Agilent GC 7890B gas chromatography coupled with an Agilent 5977B mass spectrometer detector (MSD). In the gas chromatographic system, an HP-5MS capillary column 30 m × 0.25 mm × 0.25 µm (Agilent Technologies, Santa Clara, CA, USA) was used. The GC was equipped with a split/splitless injector and an SPME inlet (Supelco, Bellefonte, PA, USA), which operated under the splitless mode during the analysis. The injection temperature of the GC inlet was 270 °C. Helium was used as a carrier gas at a constant flow of 1.2 mL/min. The oven temperature program was 60 °C for 2 min and increased at a rate of 7 °C/min to 200 °C, 5 °C/min to 300 °C, and finally at a rate of 50 °C/min to 320 °C before being held for 3 min with a total run time of 45.4 min. The MSD transfer line, ion source and quad-pole temperatures were 300, 230 and 150 °C, respectively. Ionization energy was 70 eV, and scan acquisition mode was performed at a scan ranged from 50 to 600 *m*/*z* at a scan speed of 10,000 amu/s.

### 2.4. The Extraction and Analytical Procedures

#### 2.4.1. Preparation of Diluted Standards

All the fatty acid external standard chemicals were prepared by dilution with acetonitrile to 1 mg (standards)/g (acetonitrile) in 20 mL clear glass vials. The final fatty acid standard was prepared by mixing the diluted individual standards. This included a range of fatty acids (palmitic acid, linolenic acid, linoleic acid, oleic acid and arachidic acid), p-benzoquinone, 2-methyl-p-benzoquinone, and cholesterol with final concentrations of 0.1, 0.5, 1, 10 and 100 µg/g, respectively. The second standard mixture was prepared by mixing n-alkane standards C7 to C40 (1000 mg/mL in hexane, Supelco, Bellefonte, PA, USA) to obtain concentrations of 0.1, 0.5, 1, 5 and 10 µg/g. Meanwhile, acetonitrile was used as a blank. 

#### 2.4.2. Evaluation of the Effect of Different Solvents on DI-SPME for Extraction of Lipids from *T. castaneum*

Prior to extraction, all the insects used in this research were cleaned by letting them move on a wet tissue paper for 15 min and then transferring them onto clean, dry tissue paper for 10 min. The cleaned insects were frozen with liquid nitrogen. To evaluate the effect of the solvent type on the extraction, twenty frozen dead adults *T. castaneum* were transferred into a 2-mL plastic microtube, to which 1.6 mL of solvent was added. Three milling balls were added, and the microtube was closed for homogenization using a Beadbug homogenizer for 1 min at 4000 revolutions per minute (rpm). The extracts were centrifuged at 8150× *g* for 3 min using the Dynamica mini centrifuge, and then 1.5 mL supernatant was transferred to a 2 mL HPLC clear vial using a 1000 μL micropipette. Each of the nine solvents was replicated four times.

The SPME fiber was inserted into the extract for 14 h at 25 ± 2 °C. Immediately after completing the extraction, the fiber was withdrawn and injected directly into the GC–MS injector for the determination of lipids.

#### 2.4.3. Comparison of Lipid Compositions between Two Insect Species in Acetonitrile

The adult insects were cleaned as described above. For the extraction of cuticular lipids, the cleaned insects (20 *T. castaneum* and 25 *R. dominica*) were separately transferred into 2 mL microtubes containing 1.6 mL of acetonitrile (HPLC grade, Fisher Scientific, Glee, Belgium) using a small clean brush and then sealed with screw cap. The microtubes were shaken gently by hand for 3 min, and then the extract was transferred into a 2 mL amber GC vial with septa using a micropipette.

The lipids in the remaining extracted insect bodies were homogenized using a Beadbug homogenizer in a 2 mL BeadBug™ microtube containing 1.6 mL of HPLC grade acetonitrile for 1 min at 4000 rpm before being then centrifuged at 8150× *g* for 3 min using the Dynamica mini centrifuge. The supernatant (1.5 mL) was transferred into a 2 mL amber GC vial with septa.

Each extraction with acetonitrile was repeated four times. The SPME fiber was inserted into the extract for 14 h at 25 ± 2 °C for DI-SPME extraction and the determination of the lipids by GC–MS.

### 2.5. Data Processing and Analysis

The GC–MS signals were collected by the MassHunter Acquisition software (Agilent Technologies, Santa Clara, CA, USA). The Automatic Mass Spectral Deconvolution and Identification System (AMDIS-32) software and the National Institute of Standards and Technology (NIST) mass spectra library (version 2.2) were used to identify chemical compounds. The Kovat’s retention index was used to assist identification. Data sorting and linear regression were processed by Microsoft Excel 2016. The averages of the compound areas were statistically analyzed by Metaboanalyst 4.0 (http://www.metaboanalyst.ca/faces/upload/StatUploadView.xhtml), using hierarchical cluster analyses (heatmap) [20]. Samples were uploaded to Metaboanalyst 4.0 as columns (unpaired); data filtering was characterized using the mean intensity value. Sample normalization, data transformation and data scaling were specified as a “NONE” mode. Heatmap parameters were as follows: distance measure = Euclidean; clustering algorithm = ward; and standardization = auto scale feature. The heatmap statistical model was the t-test/ANOVA. The LOD was calculated by the linear regression method [21] using Equation (1):

LOD = 3S/b(1)

where S is the standard deviation of the linear response of the GC–MS and b is the slope of the calibration curve.

## 3. Results and Discussion

### 3.1. Effect of Direct Immersion on SPME Extraction in Solvent

**Figure 1:**
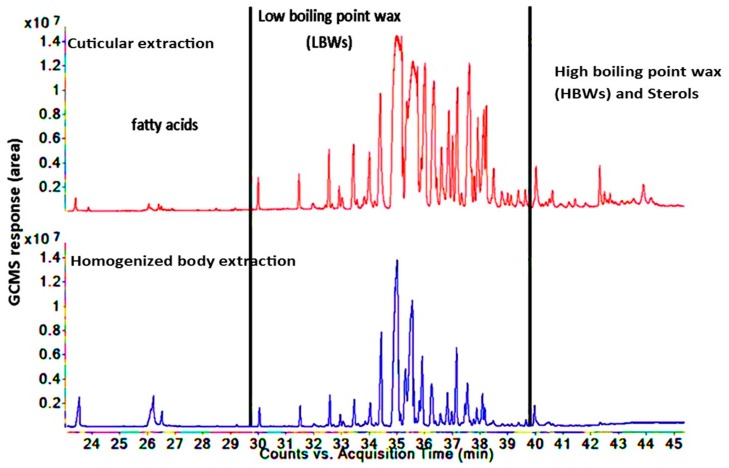
Total signal chromatogram of lipids of cuticular extraction followed by homogenized body extraction of same insects of *Tribolium castaneum*, using acetonitrile as solvent.

**Figure 2:**
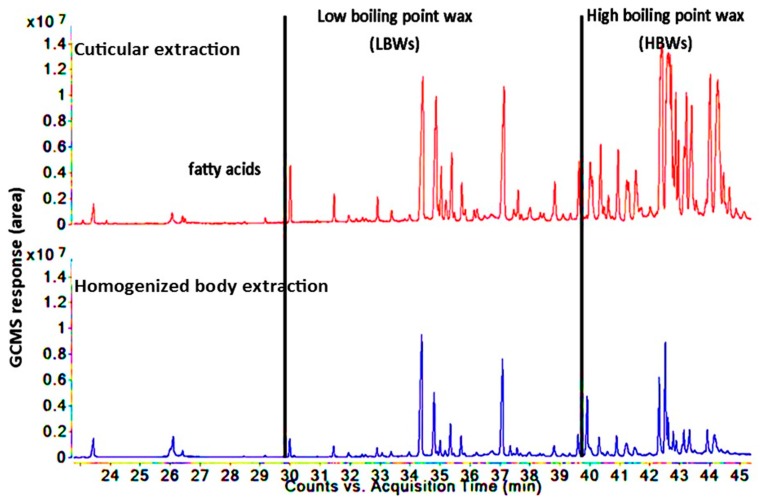
Total signal chromatogram of lipids of cuticular extraction followed by homogenized body extraction of same insects of *Rhyzopertha dominica*, using acetonitrile as solvent.

This research reported a novel comprehensive DI-SPME method for the extraction of lipids such as fatty acids and hydrocarbon waxes from the cuticular surface and the whole body of insects (Figure 1 and Figure 2). Since fiber coating was reported as a vital factor in the development of an appropriate SPME method [22,23,24], a three phase combination DVB/CAR/PDMS fiber was used in this study to extract the lipid compounds from the samples. The selection was based on previous research [25,26] and also because the fiber coating of DVB/CAR/PDMS covers wide range of polarities from non-polar to polar compounds, which enables it to extract a wide range of compounds; as such, the extraction was strongly affected by the polarity of the SPME [24]. The lipid profile of *T. castaneum* showed the most GC peaks of the hydrocarbon wax components in the low boiling point waxes (LBWs) range. In contrast, for *R. dominica,* a limited level of hydrocarbon wax peaks were seen in the same zone; however, most of the hydrocarbon wax peaks were shown in the region of high boiling point waxes (HBWs) (>300 °C of the GC–MS oven program). Lockey, 1978 [27] reported that various classes of n-alkanes and branched alkanes in *T. castaneum* were in the region of C25–C32 using conventional solvent method for lipid extraction, followed by an analysis with GC–MS. However, a similar result was simply achieved using DI-SPME fibers, e.g., alkanes and branched alkanes ranging between C25 to C32 from *T. castaneum* and C26–C34 from *R. dominica*. The DI-SPME method developed from this research preserved all the extracted insect lipids, as the extraction procedure was conducted at room temperature without the application of external heat. Since the method was direct immersion in the extract solution, there was no need to reduce the extract volume for pre-concentration, which significantly reduced the loss of some volatiles during vaporization. Moreover, an important innovation is that the DI-SPME enabled a lipid analysis without introducing additional reactions and chemicals for derivatizing lipids. This significantly led to reduced time consumption, cost, loss of lipids, and soaping. Soaping is a problem if the reaction is not carried out in very restrict conditions [28]. The DI-SPME allowed for the conduction of longer periods such as a 14 h extraction at room temperature, which resulted in absorbing most of the analytes from the sample matrixes on the SPME fiber coating. However, a previous study used SPME to extract the lipids in the headspace of *Bagrada hilaris* at high extraction temperatures of 130 and 150 °C [19], which significantly affected the distribution constant of the volatiles between the headspace and the matrix [29] and can also lead to the degradation of the long chains to small chains compounds. Roux et al., 2002 [18], used SPME to directly rub the insect cuticle layer, but rubbing cannot extract all the compounds in insect bodies.

### 3.2. Effect of Extraction Solvents

**Figure 3:**
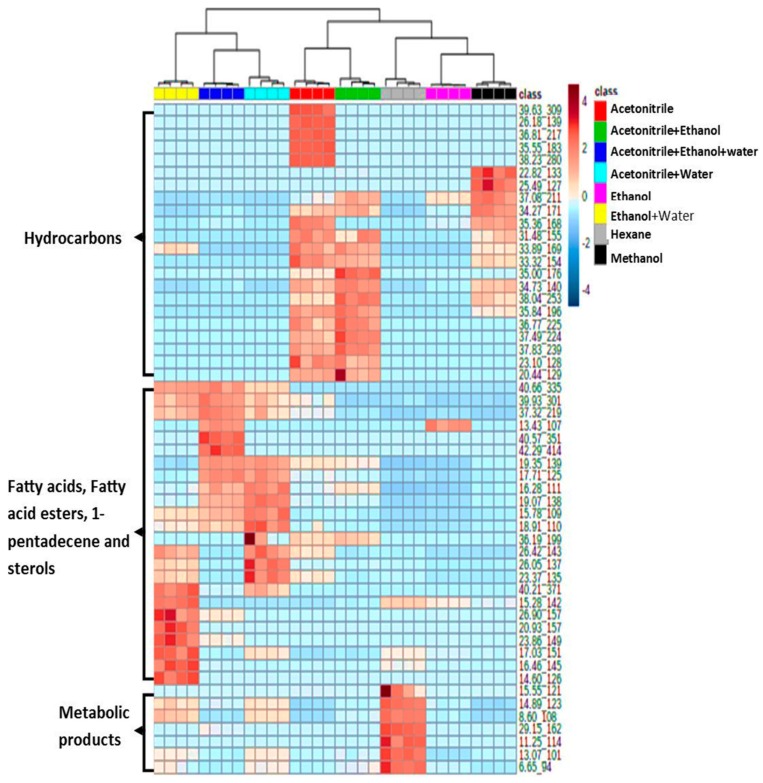
Hierarchical cluster analysis (heatmap) represents the data obtained from *T. castaneum* using eight solvents. Hot color means high gas chromatography (GC) response based on peak areas. The top dendrogram indicates the similarity among the solvents as well as the data variation among the replicates. The side arrows show that the majority of compounds were detected with high a GC response in each solvent.

Figure 3 shows the hierarchical cluster analysis (heatmap) in the form of a dendrogram. The heatmap indicates the relative intensity of four biological replicates which depended on the peak area of each compound in each solvent. Chloroform is not included in the figure because the SPME fiber coating was dissolved in this solvent. The highest relative GC response of the compound in comparison to the other was specified a “hot” color, while those that are lower in their values were given a “cold” color. The top of the dendrogram indicates the similarity among the solvents and the data variation among the replicates (Figure 3). The side arrows show a major compound with high area GC response in each solvent (Figure 3). The compounds obtained from the eight solvents consisted of an assortment of fatty acids, hydrocarbon waxes, and sterols in addition to some metabolic products, such as p-benzoquinone and methyl-p-benzoquinone (Supplementary Material Table S1). Fifty-three compounds were acquired from a total of eight tested solvents. Acetonitrile and the combination of acetonitrile and ethanol had the highest peak numbers with 41 and 34 compounds, respectively, including most of the fatty acids and hydrocarbon waxes, while the lowest number of compounds (22) (Table 1) was obtained from acetonitrile and water. It was observed that each compound had a different GC response according to the solvent used in the extraction process. Both acetonitrile and the combination of acetonitrile and ethanol showed a similar influence regarding the GC response of compounds. Most of the highest molecular weight compounds had higher intensities when acetonitrile and the combination of acetonitrile and ethanol were used as solvents. On the other hand, both the combination of acetonitrile and ethanol and water and the combination of acetonitrile and water had parallel effects (Figure 3). This could have been because of the similar polarity of the combined solvents. However, a higher GC response of some main fatty acids was detected in the combination of acetonitrile and water, such as palmitic acid (23.37_135), stearic acid (26.42_143) and linolenic acid (26.05_137) (Figure 3 and Supplementary Material Table S1) because these are more polar compounds and the presence of water in a solvent increase the polarity of the solvent.

While the sterol compounds showed a higher GC response under the acetonitrile and ethanol and water solvent, methanol and ethanol were quite similar in their extraction abilities, although methanol efficiently extracted most of the high boiling point hydrocarbon waxes. However, the use of methanol as an extraction solvent may form artificial methyl esters of fatty acids in the presence of some organic or inorganic materials [30]. As per Figure 3, with hexane, most of the compounds were between retention time (RT) = 6.65 to 15.55 min and not at higher RT where C25–C34 compounds for *Tribolium* and *Rhyzopertha* could be seen, though hexane was commonly used as a solvent to extract the cuticular lipids from many insects [13,31]. However, a low yield of hydrocarbon waxes in this study could have be due to the fact of the incompatibility of fiber coating and the solvent as a result a lack of the efficient distribution of analytes between the fiber and the solvent. Chloroform is another solvent which has been successfully used as a single solvent [32] or combined with another solvent such as hexane [16] to extract insect lipids, producing a high yield [1]. However, this was not appropriate to use for the DI-SPME technique as it can destroy the SPME fiber coating.

From the above results, acetonitrile was selected as the optimal solvent in the validation study. In previous studies, many solvents were used to extract either fatty acids or hydrocarbons, such as petroleum ether with dichloromethane [33,34], hexane with chloroform [35] and dichloromethane alone [15]. However, only acetonitrile demonstrated the ability to extract both fatty acids and hydrocarbon waxes from insects.

The results obtained from insect samples were compared with external standards of n-alkane, fatty acids, sterol, p-benzoquinone, methyl-p-benzoquinone and 1-pentadecene. The chemical compounds were identified using the NIST database after comparison with the mass spectra and retention indexes (RI) by running the external standards. The results of the LOD in Table 2 indicate that the new method could detect quinones (p-benzoquinone, 2-methyl-), fatty acids (arachidic acid), cholesterol and alkanes (heptacosane) at levels of 0.36, 0.65, 034 and 0.08 µg/g respectively. Therefore, this method has been demonstrated to be a robust method to analyze a variety of lipids.

### 3.3. Comparison of Lipid Compositions between Two Insect Species

The GC responses for *T. castaneum* and *R. dominica* that were identified as compounds from the total GC responses were 91.93 ± 2.74% and 82.22 ± 3.06% from the homogenized body and 93.38 ± 3.93% and 81.32 ± 2.82% from the cuticular extractions, respectively. Thirty-eight and 39 compounds were obtained from the cuticular and homogenized body extraction of *T. castaneum*, whereas 30 and 26 compounds were identified from the cuticular and homogenized body extraction of *R. dominica,* respectively (Table 3 and Table 4). The carbon chain lengths of *T. castaneum* varied from 25 (pentacosane) to 32 (dotriacontane) carbons, and *R. dominica* had a range of compounds from 26 (11-methylpentacosane) to 34 (tetratriacontane) carbons. According to the cuticular and homogenized body extractions, n-alkanes and methyl-branched alkanes were the major compounds identified from *R. dominica* and *T. castaneum.* A previous study by Lockey, 1978 [27] also reported the same classes of n-alkanes and branched alkanes in *T. castaneum* in the region of C25–C32. In this study, the two major lipid compounds from *T. castaneum* cuticular extraction were 11-methylheptacosane (20.71%) and 3-methylheptacosane (12.37%), and the two major lipid compounds from the homogenized body were 1-pentadecene (22.70%) and 11-methylheptacosane (16.50%). The lipid compounds 10-methyldotriacontane (14.0%) and 15-methyltritriacontane (9.93%) were the two major compounds in the cuticular extraction from *R. dominica*, and 13-methylnonacosane and 13-methylheptacosane had the highest peak areas (20.30% and 18.10%, respectively) in the homogenized body. These results demonstrate that this method could extract and identity specific hydrocarbons from different insect species. This might indicate that the method can be used as a tool for the identification of insect species, which further supports previously reported studies that used cuticular hydrocarbons as chemotaxonomic tools for the identification of insect species [36,37].

The results from both insect species showed that the majority of hydrocarbon waxes were in abundance in the cuticular extraction in comparison to the homogenized body extraction (RT = 31.5 to 39.6 min for *T. castaneum* and RT = 31.5 to 45.2 min for *R. dominica*, Figure 1 and Figure 2) including the major compounds such as 11-methylheptacosane and 3-methylheptacosane from *T. castaneum*. This is evident from the distribution coefficient between the homogenized body and the cuticular extraction (Table 3 and Table 4), where the total peak areas were 29.11% and 24.17%, respectively, for the two major compounds of 11-methylheptacosane and 3-methylheptacosane in the homogenized body as compared to the cuticular extraction, suggesting the fact that these compounds are more in abundance in the cuticular extraction. A similar result for *R. dominica* was also observed for the two major compounds 10-methyldotriacontane and 15-methyltritriacontane, which showed distribution coefficients of 10.61% and 7.11% in the homogenized extraction compared to the cuticular extraction. However, the fatty acids peak areas were opposite in the GC response. The peak areas of most of fatty acids were higher in the homogenized body extraction in comparison to the cuticular extraction. Linolenic acid showed the highest distribution coefficients of 96.01% and 88.72% for *T. castaneum* and *R. dominica,* respectively, in the homogenized body extraction. Thus, this research provides a robust tool not only to analyze cuticular and whole body lipids but to also assist in understanding the cuticular lipid compositions in comparison to internal lipid composition. This, in turn, may provide information to deduce the essential roles of lipids in many chemical and biological processes such as protecting insect bodies from dryness and pathogens [1,2,38]. 

## 4. Conclusions

The DI-SPME method coupled with GC–MS was explored for the first time to analyze insect cuticular and homogenized body lipids including hydrocarbons and fatty acids without derivatization. The four solos and their four combination solvents were evaluated, and acetonitrile was found to be the optimal solvent for the extraction of hydrocarbons and fatty acids from insects. The method was validated by analyzing the cuticular and internal lipids from two stored product insect species. The results indicate that the method is robust, reliable and sensitive for the extraction and identification of lipids from different species of insects.

## Figures and Tables

**Table 1:** The number of separated and identified compounds obtained in each solvent out of the total of 53 compounds from *T. castaneum*.

No.	Solvent	Compound Numbers	The Rate of Total Expected Compounds (%)	Number of Compounds Only Detected in Solvent
1	Acetonitrile	41	77.4	5
2	Hexane	23	43.4	1
3	Ethanol	25	47.2	0
4	Methanol	28	52.8	1
5	Acetonitrile and ethanol (1:1 *v*/*v*)	34	64.2	0
6	Acetonitrile and water (1:1 *v*/*v*)	22	41.5	0
7	Ethanol and water (1:1 *v*/*v*)	27	50.9	0
8	Ethanol and acetonitrile and water (2:2:1 *v*/*v*/*v*)	30	56.6	1

**Table 2:** The limit of detection (LOD) of external reference standards of high boiling point n-alkanes, fatty acids, sterol, p-benzoquinone, methyl-p-benzoquinone and 1-pentadecene using acetonitrile as the solvent.

	Chemical Standards	Formula	RT (min)	R^2^	LOD (µg/g)
1	p-benzoquinone	C_6_H_4_O_2_	4.84	0.995	0.47
2	p-benzoquinone, 2-methyl-	C_7_H_6_O_2_	6.72	0.976	0.36
3	1-pentadecene	C_15_H_30_	16.17	0.999	0.22
4	Palmitic acid	C_16_H_32_O_2_	23.44	0.990	0.84
5	Henicosane	C_21_H_44_	25.35	0.939	0.21
6	Linolenic acid	C_18_H_30_O_2_	25.99	0.937	0.87
7	Linoleic acid	C_18_H_32_O_2_	26.13	0.989	0.87
8	Oleic acid	C_18_H_34_O_2_	26.43	0.993	0.89
9	Docosane	C_22_H_46_	26.60	0.964	0.13
10	Arachidic acid	C_20_H_40_O_2_	28.21	0.963	0.65
11	Tricosane	C_23_H_48_	31.30	0.961	0.13
12	Tetracosane	C_24_H_50_	32.24	0.920	0.21
13	Pentacosane	C_25_H_52_	33.06	0.923	0.24
14	Hexacosane	C_26_H_54_	34.41	0.961	0.14
15	Heptacosane	C_27_H_56_	35.27	0.988	0.08
16	Octacosane	C_28_H_58_	36.10	0.979	0.10
17	Nonacosane	C_29_H_60_	37.40	0.987	0.08
18	Triacontane	C_30_H_62_	39.52	0.988	0.08
19	Hentriacontane	C_31_H_64_	41.48	0.982	0.09
20	Cholesterol	C_27_H_46_O	39.98	0.948	0.34
21	Dotriacontane	C_32_H_66_	42.45	0.935	0.26

RT is retention time, LOD is the limit of detection, and R^2^ is a linear regression coefficient. Based on solvent weight, the concentration of standards presents as µg/g.

**Table 3:** Extracted and identified compounds from the cuticle layer and homogenized body of *T. castaneum* in acetonitrile.

Compounds	RT (min)	NIST RI	Calculated RI	Qualitative *m/z*	GC Response (10^5^) ± SD, n = 4		Relative GC Response		Distribution Coefficient B/(A + B) × 100
					Cuticular Extraction (a)	Homogenized Body Extraction (b)	Cuticular	Homogenized Body	
2-methylbenzoquinone	6.73	1116	1117	122	14 ± 1	29 ± 39	0.13	0.49	66.64
2-ethyl-p-benzoquinone	8.66	1215	1212	108	47 ± 8	46 ± 4	0.41	0.78	49.27
1,4-benzenediol, 2-methyl-	13.55	1223	1234	124	16 ± 3	65 ± 2	0.14	1.1	80.52
1,2-benzenediol, 4-ethyl-	15.07	1392	1388	138	30 ± 3	105 ± 2	0.26	1.78	77.65
7-dodecenol	15.83	1465	1468	165	72 ± 12	84 ± 4	0.63	1.43	53.86
1-pentadecene	16.26	1502	1504	154	1250 ± 172	1336 ± 4	10.98	22.70	51.66
Benzene, 1-ethoxy-4-isothiocyanato-	16.54	1527	1528	166	2 ± 0.3	2 ± 0.1	0.02	0.03	43.78
1-(2-hydroxy-4-methoxyphenyl)propan-1-one	17.07	1538 *	1558	151	47 ± 2	33 ± 3	0.40	0.56	41.42
7-hexadecene, (Z)-	17.82	1620	1605	152	39 ± 1	28 ± 3	0.34	0.47	41.75
1,8,11-heptadecatriene, (Z,Z)-	18.98	1655	1653	149	ND	11 ± 0.4	ND	0.19	100
cis-7-tetradecen-1-ol	19.14	1660	1661	179	515 ± 60	357 ± 31	4.52	6.06	40.9
2-hexadecanol	19.50	1702	1705	182	453 ± 36	374 ± 17	3.98	6.37	45.23
Myristic acid	20.44	1752	1755	185	ND	0.82 ± 0.09	ND	0.01	100
Palmitoleic acid	23.10	1936	1938	192	4 ± 0.3	ND	0.04	ND	0
Palmitic acid	23.60	1954	1956	199	17 ± 2	143 ± 12	0.15	2.44	89.54
Linolenic acid	26.15	2115	2119	222	3 ± 0.2	72 ± 5	0.03	1.23	96.01
Oleic acid	26.25	2134	2125	220	ND	92 ± 4	ND	1.57	100
Stearic acid	26.57	2153	2157	227	5 ± 0.6	45 ± 5	0.05	0.77	89.68
Unknown	31.52	-	2505	-	12 ± 2	4 ± 0.4	0.10	0.08	26.71
Pentacosane	33.30	2500	2515	238	33 ± 3	14 ± 0.7	0.29	0.24	30.49
Hexacosane	33.85	2600	2612	266	174 ± 27	59 ± 3	1.53	1.00	25.3
Unknown	34.06	-	2618	-	27 ± 2	38 ± 4	0.23	0.65	58.52
2-methylhexacosane	34.28	2661	2684	294	105 ± 10	5 ± 0.7	0.92	0.08	4.418
13-methylheptacosane	34.41	2731	2741	296	289 ± 22	274 ± 19	2.53	4.66	48.72
11-methylheptacosane	34.82	2734	2750	309	2358 ± 186	969 ± 85	20.71	16.5	29.11
2-methylheptacosane	35.02	2762	2766	336	635 ± 37	218 ± 14	5.57	3.71	25.57
3-methylheptacosane	35.58	2773	2771	337	1409 ± 147	449 ± 17	12.37	7.64	24.17
Octacosane	35.87	2800	2815	323	540 ± 35	118 ± 10	4.74	2.00	17.9
3-methyloctacosane	36.28	2872	2849	351	531 ± 76	133 ± 10	4.66	2.27	20.05
Nonacosane	36.60	2900	2902	365	177 ± 29	30 ± 3	1.55	0.51	14.58
Unknown	36.85	-	2908	-	165 ± 18	41 ± 3	1.44	0.70	19.99
Unknown	37.08	-	2911	-	437 ± 15	393 ± 31	3.83	6.68	47.36
Unknown	37.49	-	2917	-	457 ± 40	23 ± 2	4.01	0.40	4.86
13-methylnonacosane	37.58	2930	2927	379	766 ± 74	71 ± 7	6.73	1.21	8.52
11-methylnonacosane	37.92	2939	2950	393	31 ± 2	ND	0.27	ND	0
Nonacosane, 2-methyl-	38.13	2962	2961	421	229 ± 16	79 ± 2	2.42	1.34	25.56
3-methylnonacosane	38.44	2974	2973	395	275 ± 17	10 ± 1	2.41	0.16	3.351
Triacontane	39.64	3000	3003	239	141 ± 17	13 ± 1	1.24	0.22	8.425
Cholesterol	40.20	3087	3060	386	29 ± 1	75 ± 6	025	1.29	73.01
Desmosterol	40.51	3125	3133	364	15 ± 2	29 ± 1	0.13	0.49	66.06
Dotriacontane	42.37	3200	3203	449	36 ± 1	13 ± 1	0.32	0.22	26.27

The list contains only the compound that was identified properly; some compound may be present on the GC–MS chromatogram but are not on the list due to the lack of the identification. Compounds with matching RI differences more than 30 were reported as “Unknown.” RT = retention time; NIST RI = retention indices obtained from National Institute of Standards and Technology database (NIST). * Estimated non-polar retention index (n-alkane scale NIST). Calculated RI = retention indices calculated using n-alkane standards C7–C40. Relative areas were calculated according to the total area of the listed compounds. *m*/*z* = mass to charge ratio. SD = standard deviation (n = 4). ND = not detected.

**Table 4:** Extracted and identified compounds from cuticle layer and homogenized body of *R. dominica* in acetonitrile.

Compounds	RT (min)	NIST RI	Calculated RI	Qualitative M/Z	GC Response (10^5^) ± SD, n = 4		Relative GC Response		Distribution Coefficient B/(A + B) × 100
					Cuticular Extraction (A)	Homogenized Body Extraction (B)	Cuticular	Homogenized Body	
Palmitic acid	23.53	1954	1956	199	21 ± 3	ND	0.53	ND	0
Linolenic acid	26.06	2115	2119	222	6 ± 0.7	51 ± 5	0.17	4.39	88.72
Stearic acid	26.42	2153	2157	227	3 ± 0.3	13 ± 3	0.07	1.15	83.79
Octadecanamide, N-(2-hydroxyethyl)-	29.16	2347	2347	280	17 ± 0.7	9 ± 1	0.43	0.75	34.31
Unknown	31.46	-	2515	-	17 ± 3	ND	0.45	ND	0
11-methylpentacosane	31.94	2535	2555	281	5 ± 0.8	3 ± 0.2	0.12	0.28	41.64
Unknown	32.91	-	2628	-	17 ± 2	ND	0.45	ND	0
13-methylheptacosane	34.41	2731	2741	296	291 ± 56	209 ± 26	7.52	18.10	41.77
2-methylheptacosane	34.98	2762	2766	336	166 ± 13	31 ± 6	4.28	2.66	15.64
3-methylheptacosane	35.34	2773	2771	337	154 ± 28	7 ± 1	3.98	0.59	4.20
Octacosane	35.82	2800	2815	323	45 ± 4	33 ± 7	1.16	2.89	42.77
Unknown	37.10	-	2912	-	321 ± 25	15 ± 2	8.30	1.32	4.54
13-methylnonacosane	37.58	2930	2927	379	33 ± 3	234 ± 21	0.84	20.3	87.79
Triacontane	39.64	3000	3003	239	156 ± 30	16 ± 2	4.04	1.44	9.59
Cholesterol	40.00	3087	3060	386	12 ± 1	48 ± 8	0.32	4.21	79.67
Hentriacontane	41.22	3100	3117	435	65 ± 3	23 ± 3	1.69	1.98	25.93
2-methylhentriacontane	41.53	3162	3152	436	68 ± 5	28 ± 5	1.77	2.46	29.39
3-methylhentriacontane	42.01	3172	3182	424	29 ± 6	14 ± 3	0.75	1.21	32.50
Dotriacontane	42.37	3200	3203	449	271 ± 51	6 ± 0.9	7.00	0.52	2.16
10-methyldotriacontane	42.50	3235	3218	477	540 ± 57	64 ± 7	14.00	5.55	10.61
8-methyldotriacontane	42.60	3240	3221	450	92 ± 6	118 ± 9	2.37	10.20	56.17
Unknown	42.78	-	3231	-	181 ± 12	17 ± 2	4.69	1.46	8.53
Unknown	42.87	-	3237	-	66 ± 12	ND	1.70	ND	0
Unknown	42.96	-	3249	-	243 ± 36	24 ± 4	6.29	2.08	8.98
Dotriacontane, 2-methyl-	43.22	3263	3266	481	260 ± 38	30 ± 5	6.29	2.64	10.47
Unknown	43.40	-	3276	-	240 ± 20	42 ± 5	6.22	3.66	14.95
15-methyltritriacontane	44.03	3333	3323	463	384 ± 60	29 ± 5	9.93	2.55	7.11
Unknown	44.47	-	3351	-	58 ± 6	63 ± 12	1.50	5.47	52.17
Tetratriacontane	44.88	3400	3387	492	65 ± 12	11 ± 0.7	1.68	0.95	14.40

The list contains only the compound that was identified properly; some compound may be present on the GC–MS chromatogram but are not on the list due to the lack of the identification. Compounds with matching RI differences of more than 30 were reported as “Unknown.” RT = retention time; NIST RI = retention indices obtained from National Institute of Standards and Technology database (NIST). Calculated RI = retention indices were calculated using n-alkane standards C7–C40. Relative areas were calculated according to the total area of the listed compounds; *m*/*z* = mass to charge ratio; SD = standard deviation (n = 4). ND = not detected.

## Supplementary Materials

The following are available online at https://www.mdpi.com/2075-4450/10/10/363/s1, Table S1: Extracted and identified compounds from homogenized whole *T. castaneum* in different solvents.

## References

[1] Cerkowniak M., Puckowski A., Stepnowski P., Gołębiowski M. (2013). The use of chromatographic techniques for the separation and the identification of insect lipids. J. Chromatogr. B.

[2] Cohen E., Moussian B. (2016). Extracellular Composite Matrices in Arthropods.

[3] Desbois A.P., Smith V.J. (2010). Antibacterial free fatty acids: Activities, mechanisms of action and biotechnological potential. Appl. Microbiol. Biotechnol..

[4] Gibbs A.G. (1998). Water-proofing properties of cuticular lipids. Am. Zool..

[5] Gołębiowski M., Maliński E., Nawrot J., Stepnowski P. (2008). Identification and characterization of surface lipid components of the dried-bean beetle *Acanthoscelides obtectus* (Say) (Coleoptera: Bruchidae). J. Stored Prod. Res..

[6] Gołębiowski M., Boguś M.I., Paszkiewicz M., Stepnowski P. (2011). Cuticular lipids of insects as potential biofungicides: Methods of lipid composition analysis. Anal. Bioanal. Chem..

[7] Nelson D.R., Charlet L.D. (2003). Cuticular hydrocarbons of the sunflower beetle, *Zygogramma exclamationis*. Comp. Biochem. Physiol. B: Biochem. Mol. Biol..

[8] Snellings Y., Herrera B., Wildemann B., Beelen M., Zwarts L., Wenseleers T., Callaerts P. (2018). The role of cuticular hydrocarbons in mate recognition in *Drosophila suzukii*. Scie. Rep..

[9] Berson J.D., Simmons L.W. (2019). Female cuticular hydrocarbons can signal indirect fecundity benefits in an insect. Evolution.

[10] Ferveur J.-F., Cortot J., Rihani K., Cobb M., Everaerts C. (2018). Desiccation resistance: effect of cuticular hydrocarbons and water content in *Drosophila melanogaster* adults. PeerJ.

[11] Gołębiowski M., Maliński E., Boguś M.I., Kumirska J., Stepnowski P. (2008). The cuticular fatty acids of *Calliphora vicina*, *Dendrolimus* pini and *Galleria mellonella* larvae and their role in resistance to fungal infection. Insect Biochem. Mol. Biol..

[12] Toolson E.C., Kuper-Simbrón R. (1989). Laboratory evolution of epicuticular hydrocarbon composition and cuticular permeability in Drosophila pseudoobscura: effects on sexual dimorphism and thermal-acclimation ability. Evolution.

[13] Ye G., Li K., Zhu J., Zhu G., Hu C. (2007). Cuticular hydrocarbon composition in pupal exuviae for taxonomic differentiation of six necrophagous flies. J. Med. Entomol..

[14] Buckner J.S., Hagen M.M., Nelson D.R. (1999). The composition of the cuticular lipids from nymphs and exuviae of the silverleaf whitefly, *Bemisia argentifolii*. Comp. Biochem. Physiol. B Biochem. Mol. Biol..

[15] Saïd I., Costagliola G., Leoncini I., Rivault C. (2005). Cuticular hydrocarbon profiles and aggregation in four *Periplaneta* species (Insecta: Dictyoptera). J. Insect. Physiol..

[16] Buckner J.S., Pitts-Singer T.L., Guédot C., Hagen M.M., Fatland C.L., Kemp W.P. (2009). Cuticular lipids of female solitary bees, *Osmia lignaria* Say and *Megachile rotundata* (F.)(Hymenoptera: Megachilidae). Comp. Biochem. Physiol. B Biochem. Mol. Biol..

[17] Ginzel M.D., Moreira J.A., Ray A.M., Millar J.G., Hanks L.M. (2006). (Z)-9-Nonacosene—major component of the contact sex pheromone of the beetle *Megacyllene caryae*. J. Chem. Ecol..

[18] Roux E., Sreng L., Provost E., Roux M., Clement J.L. (2002). Cuticular hydrocarbon profiles of dominant versus subordinate male *Nauphoeta cinerea* cockroaches. J. Chem. Ecol..

[19] De Pasquale C., Guarino S., Peri E., Alonzo G., Colazza S. (2007). Investigation of cuticular hydrocarbons from *Bagrada hilaris* genders by SPME/GC-MS. Anal. Bioanal. Chem..

[20] Chong J., Soufan O., Li C., Caraus I., Li S., Bourque G., Wishart D.S., Xia J. (2018). MetaboAnalyst 4.0: Towards more transparent and integrative metabolomics analysis. Nucleic Acids Res..

[21] Shrivastava A., Gupta V.B. (2011). Methods for the determination of limit of detection and limit of quantitation of the analytical methods. Chron. Young sci..

[22] Alnajim I., Agarwal M., Liu T., Ren Y. (2019). A Novel Method for the Analysis of Volatile Organic Compounds (VOCs) from Red Flour Beetle *Tribolium castaneum* (H.) Using Headspace-SPME Technology. Curr. Anal. Chem..

[23] Jeleń H.H., Obuchowska M., Zawirska-Wojtasiak R., Wasowicz E. (2000). Headspace solid-phase microextraction use for the characterization of volatile compounds in vegetable oils of different sensory quality. J. Agric. Food. Chem..

[24] Balasubramanian S., Panigrahi S. (2011). Solid-phase microextraction (SPME) techniques for quality characterization of food products: a review. Food Bioproc. Tech..

[25] Qiu R., Qu D., Hardy G.E.S.J., Trengove R., Agarwal M., Ren Y. (2014). Optimization of headspace solid-phase microextraction conditions for the identification of *Phytophthora cinnamomi* rands. Plant Dis..

[26] Niu Y., Hardy G., Hua L., Trengove R., Agarwal M., Cheng H., Ren Y. Optimization of HS-SPME-GC method for detection of stored grain insects. Proceedings of the International Conference on Controlled Atmosphere and Fumigation in Stored Products.

[27] Lockey K.H. (1978). Hydrocarbons of adult *Tribolium castaneum* Hbst. and *Tribolium confusum* Duv.(Coleoptera: Tenebrionidae). Comp. Biochem. Physiol. B Comp. Biochem..

[28] Aldai N., Murray B.E., Nájera A.I., Troy D.J., Osoro K. (2005). Derivatization of fatty acids and its application for conjugated linoleic acid studies in ruminant meat lipids. J. Sci. Food Agric..

[29] Kataoka H., Lord H.L., Pawliszyn J. (2000). Applications of solid-phase microextraction in food analysis. J. Chrmatogr. A.

[30] Brondz I., Ekeberg D., Høiland K., Bell D.S., Annino A.R. (2007). The real nature of the indole alkaloids in Cortinarius infractus: Evaluation of artifact formation through solvent extraction method development. J. Chromatogr. A.

[31] Caputo B., Dani F., Horne G., N’fale S., Diabate A., Turillazzi S., Coluzzi M., Costantini C., Priestman A., Petrarca V. (2007). Comparative analysis of epicuticular lipid profiles of sympatric and allopatric field populations of *Anopheles gambiae* ss molecular forms and *An. arabiensis* from Burkina Faso (West Africa). Insect Biochem. Mol. Biol..

[32] Buckner J.S., Mardaus M.C., Nelson D.R. (1996). Cuticular lipid composition of *Heliothis virescens* and *Helicoverpa zea* pupae. Comp. Biochem. Physiol. Part B Biochem. Mol. Biol..

[33] Gołębiowski M., Boguś M.I., Paszkiewicz M., Stepnowski P. (2010). The composition of the free fatty acids from *Dendrolimus pini* exuviae. J. Insect. Physiol..

[34] Gołębiowski M., Cerkowniak M., Dawgul M., Kamysz W., BOGUŚ M.I., Stepnowski P. (2013). The antifungal activity of the cuticular and internal fatty acid methyl esters and alcohols in *Calliphora vomitoria*. Parasitology.

[35] Nelson D.R., Freeman T.P., Buckner J.S. (2000). Waxes and lipids associated with the external waxy structures of nymphs and pupae of the giant whitefly, *Aleurodicus dugesii*. Comp. Biochem. Physiol. Part B Biochem. Mol. Biol..

[36] Barbosa R.R., Braga M.V., Blomquist G.J., Queiroz M.M.d.C. (2017). Cuticular hydrocarbon profiles as a chemotaxonomic tool for three blowfly species (Diptera: Calliphoridae) of forensic interest. J. Nat. Hist..

[37] Braga M.V., Pinto Z.T., de Carvalho Queiroz M.M., Matsumoto N., Blomquist G.J. (2013). Cuticular hydrocarbons as a tool for the identification of insect species: Puparial cases from Sarcophagidae. Acta Tropica.

[38] Wang C., Leger R.J.S. (2005). Developmental and transcriptional responses to host and non-host cuticles by the specific locust pathogen *Metarhizium anisopliae* var. acridum. Eukaryot. Cell..

