# Supplementary material for: New Method of Analysis of Lipids in Tribolium castaneum (Herbst) and Rhyzopertha dominica (Fabricius) Insects by Direct Immersion Solid-Phase Microextraction (DI-SPME) Coupled with GC–MS
Source: Insects. 2019 Oct 19;10(10):363. doi: 10.3390/insects10100363 (PMC6835878; doi:10.3390/insects10100363)
Supplement: Supplementary file 1 [file insects-10-00363-s001.pdf]

Supplementary Results

Table S1. Extracted and identified compounds from homogenized whole *T. castaneum* in different solvents.

Feature ID	Chemical Name	NIST RI	Calculated RI	Qualitative M/Z	GC response (10^5) ± SD, n = 4							
					Acetonitrile	Hexane	Ethanol	Methanol	Acetonitrile + ethanol	Acetonitrile + water	Ethanol + Water	Ethanol + Acetonitrile + Water
**6.65_94**	2-methylbenzoquinone	1116	1117	122	14 ± 1	242 ± 29	25 ± 3	5 ± 0.4	41 ± 5	49 ± 3	43 ± 3	10 ± 2
**8.60_108**	2-ethyl-p-benzoquinone	1215	1212	108	18 ± 3	425 ± 24	60 ± 6	20 ± 1	86 ± 10	171 ± 9	227 ± 26	34 ± 6
**11.25_114**	3-undecanone	1283	1289	134	ND	8 ± 3	ND	ND	ND	ND	ND	ND
**13.07_101**	Methoxytoluquinone	1281	1332	128	30 ± 3	346 ± 32	ND	29 ± 2	43 ± 17	83 ± 4	70 ± 6	20 ± 0.3
**13.43_107**	1,4-benzenediol, 2-methyl-	1223	1234	124	7 ± 0.05	ND	64 ± 10	ND	ND	ND	ND	69 ± 5
**14.60_126**	2,5-cyclohexadiene-1,4-dione, 2-ethoxy-5-methyl -	1381 *	1373	132	ND	ND	ND	ND	ND	ND	5 ± 0.6	ND
**14.89_123**	1,2-benzenediol, 4-ethyl-	1392	1388	138	51 ± 10	584 ± 41	120 ± 7	44 ± 3	113 ± 9	217 ± 18	257 ± 39	137 ± 13
**15.28_142**	Ethanone, 1-(2-hydroxy-4-methoxyphenyl)-4-	1438	1432	126	ND	32 ± 0.3	16 ± 0.6	11 ± 1	4 ± 0.2	ND	71.35 ± 5.35	ND
**15.55_121**	3-hydroxybenzoic acid methyl ester	1417	1447	127	ND	204 ± 48	ND	ND	ND	ND	ND	ND
**15.78_109**	7-dodecenol	1465	1468	165	73 ± 8	17 ± 2	25 ± 3	56 ± 7	70 ± 11	565 ± 82	245 ± 18	358 ± 35
**16.28_111**	1-pentadecene	1502	1504	154	815 ± 72	207 ± 21	326 ± 27	604 ± 39	1124 ± 79	2429 ± 244	792 ± 86	1779 ± 271
**16.46_145**	Benzene, 1-ethoxy-4-isothiocyanato-	1527	1528	166	1 ± 0.1	16 ± 0.7	2 ± 0.1	ND	0.8 ± 0.04	ND	139 ± 23	5 ± 0.3
**17.03_151**	1-(2-hydroxy-4-methoxyphenyl)propan-1-one	1538 *	1558	151	41 ± 3	296 ± 9	219 ± 3	164 ± 30	78 ± 1	391 ± 51	1204 ± 171	109 ± 28
**17.71_125**	7-hexadecene, (Z)-	1620	1605	152	25 ± 3	5 ± 0.2	4 ± 0.6	12 ± 1	20 ± 2	82 ± 13	14 ± 2	94 ± 8
**18.91_110**	1,8,11-heptadecatriene, (Z,Z)-	1655	1653	149	15 ± 2	2 ± 0.1	4 ± 0.4	9 ± 0.4	9 ± 0.8	85 ± 15	24 ± 3	40 ± 4
**19.07_138**	cis-7-tetradecen-1-ol	1660	1661	179	328 ± 46	56 ± 6	104 ± 11	227 ± 11	334 ± 26	1451 ± 92	312 ± 27	945 ± 24
**19.35_139**	2-hexadecanol	1702	1705	182	370 ± 21	34 ± 5	72 ± 9	188 ± 11	317 ± 22	722 ± 58	111 ± 21	771 ± 24
**20.44_129**	Myristic acid	1752	1755	185	2 ± 0.1	ND	ND	ND	3 ± 1	ND	ND	ND
**20.93_157**	Tetradecanoic acid, ethyl ester	1794	1780	213	ND	ND	ND	ND	ND	1.23 ± 0.12	10 ± 3	ND
**22.82_133**	Hexadecanoic acid, methyl ester	1926	1934	189	ND	ND	ND	14 ± 4	0.5 ± 0.08	ND	ND	ND
**23.10_128**	Palmitoleic acid	1936	1938	192	97 ± 10	ND	ND	ND	55 ± 8	8 ± 2	ND	ND
**23.37_135**	Palmitic acid	1954	1951	199	356 ± 59	106 ± 5	12 ± 0.7	15 ± 1	77 ± 13	1272 ± 207	553.17 ± 76	39 ± 2
**23.86_149**	Palmitic acid, ethyl ester	1993	2002	201	ND	ND	5 ± 0.9	ND	ND	ND	259 ± 49	32 ± 6
**25.49_127**	Z,Z-2,13-Octadecadien-1-ol	2078	2084	206	ND	ND	ND	11 ± 2	ND	ND	ND	ND
**26.05_137**	Linolenic acid	2115	2119	222	390 ± 42	209 ± 13	10 ± 2	15 ± 2	287 ± 42	4585 ± 285	1754 ± 203	49 ± 9
**26.18_139**	Oleic acid	2134	2125	220	474 ± 6	ND	4 ± 0.4	4 ± 0.2	ND	5 ± 0.6	8 ± 0.1	ND
**26.42_143**	Stearic acid	2153	2157	227	188 ± 26	34 ± 2	4 ± 0.1	4 ± 0.2	23 ± 4	475 ± 59	367.74 ± 15	15 ± 2

**26.90_157**	Stearic acid, ethyl ester	2195	2194	241	ND	ND	1 ± 0.1	ND	ND	ND	80 ± 14	14 ± 2
**29.15_162**	Octadecanamide, N-(2-hydroxy-ethyl)-	2347	2355	238	ND	61 ± 4	ND	ND	ND	ND	ND	4 ± 0.6
**31.48_155**	Unknown	-	2505	-	15 ± 2	ND	ND	7 ± 1	11 ± 3	ND	ND	ND
**33.32_154**	Pentacosane	2500	2515	238	55 ± 5	ND	ND	24 ± 3	35 ± 3	ND	ND	ND
**33.89_169**	Hexacosane	2600	2612	266	48 ± 7	ND	ND	20 ± 2	44 ± 9	ND	23 ± 3	ND
**34.27_171**	2-methylhexacosane	2661	2684	294	69 ± 8	3 ± 0.1	24 ± 2	121 ± 16	84 ± 8	11 ± 1	6 ± 0.6	16 ± 2
**34.73_140**	11-methylheptacosane	2734	2750	309	385 ± 50	8 ± 1	89 ± 12	501 ± 42	864 ± 84	ND	ND	71 ± 11
**35.00_176**	2-methylheptacosane	2762	2766	336	1153 ± 13	ND	13 ± 1	18 ± 2	1709 ± 174	ND	ND	13 ± 2
**35.36_168**	Unknoun	-	2771	337	343 ± 46	ND	51 ± 8	263 ± 20	ND	ND	ND	27 ± 5
**35.55_183**	3-methylheptacosane	2773	2771	337	636 ± 18	ND	ND	ND	ND	ND	ND	ND
**35.84_196**	Octacosane	2800	2815	323	184 ± 21	ND	ND	61 ± 11	218 ± 27	ND	ND	ND
**36.19_199**	3-methyloctacosane	2872	2849	351	174 ± 22	ND	ND	ND	198 ± 23	209 ± 57	ND	ND
**36.77_225**	Nonacosane	2900	2902	365	33 ± 8	ND	ND	ND	47 ± 9	ND	ND	ND
**36.81_217**	Unknown	-	2908	-	33 ± 0.7	ND	ND	ND	ND	ND	ND	ND
**37.08_211**	Unknown	-	2911	-	50 ± 3	16 ± 2	90 ± 11	240 ± 43	162 ± 22	ND	ND	7 ± 0.8
**37.32_219**	Unknown	-	2917	-	23 ± 0.7	ND	5 ± 0.5	ND	7 ± 1	44 ± 10	52 ± 1	84 ± 9
**37.49_224**	13-methylnonacosane	2930	2927	379	72 ± 9	ND	ND	ND	138 ± 31	ND	ND	ND
**37.83_239**	11-methylnonacosane	2939	2950	393	38 ± 2	ND	ND	ND	57 ± 9	ND	ND	ND
**38.04_253**	Nonacosane, 2-methyl-	2962	2961	421	40 ± 4	ND	ND	49 ± 7	114 ± 12	ND	ND	ND
**38.23_280**	3-methylnonacosane	2974	2973	395	37 ± 0.5	ND	ND	ND	ND	ND	ND	ND
**39.63_309**	Triacontane	3000	3003	239	21 ± 2	ND	ND	ND	ND	ND	ND	ND
**39.93_301**	Cholesterol	3087	3060	386	88 ± 10	28 ± 3	11 ± 0.7	15 ± 1	12 ± 2	142 ± 18	189 ± 37	255 ± 36
**40.21_371**	Unknown	-	3075	-	ND	ND	ND	ND	ND	25 ± 3	50 ± 7	4 ± 0.2
**40.57_351**	Unknown	-	3129	-	ND	ND	ND	ND	ND	ND	ND	8 ± 2
**40.66_335**	Desmosterol	3125	3133	364	0.9 ± 0.2	ND	ND	ND	ND	8 ± 0.2	13 ± 0.6	14 ± 2
**42.29_414**	Dotriacontane	3200	3203	449	0.9 ± 0.01	ND	ND	ND	ND	ND	ND	15 ± 2

Feature ID includes retention time (min) and mass to charge ratio m/z. Compounds with match RI difference more than 30 were reported as "Unknown"; *Estimated non-polar retention index (n-alkane scale NIST); SD=standard deviation (n = 4). ND=not detected.
